# Bio-logging, new technologies to study conservation physiology on the move: a case study on annual survival of Himalayan vultures

**DOI:** 10.1007/s00359-017-1180-x

**Published:** 2017-06-13

**Authors:** Sherub Sherub, Wolfgang Fiedler, Olivier Duriez, Martin Wikelski

**Affiliations:** 1 Ugyen Wangchuck Institute for Conservation and Environment Research, Lamai Goempa, Bumthang, Bhutan; 2 Department of Migration and Immuno-Ecology, Max-Planck Institute of Ornithology, Am Obstberg 1, 78315 Radolfzell, Germany; 3 Ornithology, Konstanz University, 78457 Constance, Germany; 4 Centre d’Ecologie Fonctionnelle et Evolutive, UMR 5175 CNRS-Université de Montpellier-EPHE-Université Paul Valery, 1919 Route de Mende, 34293 Montpellier Cedex 5, France

**Keywords:** Conservation physiology, Migration, Annual movement, Survival, Environmental parameters

## Abstract

Bio-logging, the on-animal deployment of miniaturised electronic data recorders, allows for the study of location, body position, and physiology of individuals throughout their ontogeny. For terrestrial animals, 1 Hz GPS-position, 3D-body acceleration, and ambient temperature provide standard data to link to the physiology of life histories. Environmental context is added at ever finer scales using remote sensing earth observation data. Here we showcase the use of such bio-logging approaches in a conservation physiology study on endangered Himalayan vultures (*Gyps himalayensis*). We determine environmental, behavioural, and physiological causes of survival in immature birds that roam from wintering sites in India, Bhutan, and Nepal towards summer areas in Tibet and Mongolia. Five of 18 immature griffons died during one year. Individuals that died had failed to migrate sufficiently far northward (>1500 km) in spring. Individuals likely died if they flew against headwinds from the north or were less able to find thermal updrafts. Surviving individuals migrated to cold and dry areas with low population density. We highlight flight experience, long distance movements, and remote places with low human population as factors critical for the survival of Himalayan vultures. High-resolution bio-logging studies can advance conservation management by pinpointing where and why migratory animals have problems and die.

## Introduction

Physiological investigations of animals can now increasingly ‘go wild’ with the rapid advancement of ever smaller electronic bio-logging units (Block 2005; Ropert-Coudert and Wilson 2005; Wilson et al. 2008, 2014, 2015; Bridge et al. 2011). Such investigations into the exact details and physiological mechanisms underlying life history or population processes are increasingly important today in light of widespread, global population declines of many animal species (Wikelski and Cooke 2006; Morales et al. 2010; Cooke et al. 2014; Lennox et al. 2016). Researchers in the bio-logging field deploy animal-attached micro-electronic units (‘tags’) that record behaviour, movement, physiology, and environment while an individual is conducting its day-to-day activities, potentially leading to a golden age in ecology and physiological ecology (Kays et al. 2015; Wilmers et al. 2015).

State-of-the-art bio-logging tags include many different sensors and record from them with a sufficiently high sampling rate to allow for a near-complete reconstruction of an individual’s 3D-path through its environment, its movement characteristics, behavioural changes as well as a dynamic assessment of its internal physiological state (Hawkes et al. 2013; Bishop et al. 2015; Scott et al. 2015). Usually, the on-board power supply is too weak and the memory capacity of the bio-logging tag is too small to record high-definition data continuously, thus, timed sub-sampling is used to gain a statistically valid picture of an individual life (Holland et al. 2009; Shamoun-Baranes et al. 2012). Commonly used sensors report GPS position, 3D-accelerometry, light-level information, conductivity, salinity, external as well as body temperature, heart rate or neuro-state (Rattenborg et al. 2008a; Nathan et al. 2012). As these sensors have different power requirements, not all of them can be recorded at the same rate. The most power hungry sensor, GPS positioning, is often scheduled either using a ‘behavioural’ 3D-acceleration trigger, i.e., only starts when the animals become very active or when it starts beating its wings (Brown et al. 2012; LaPoint et al. 2013). Alternatively, GPS sensing is scheduled whenever the battery power is sufficiently high to allow for high-definition reporting, usually at 1 Hz intervals (Flack et al. 2016), but reaching up to 10 Hz (Bouten et al. 2013).

One of the most powerful and simple sensors for conservation physiologists, 3D-acceleration, has not yet reached its full analytical potential (Wilson et al. 2006; Shepard et al. 2008; Gleiss et al. 2011; Nathan et al. 2012). 3D-acceleration sensors can easily be attached externally, e.g., in a leg band or an ear tag, and can be calibrated against behaviour either on a population or (ideally) individual level (Nathan et al. 2012; Brown et al. 2013). Combined with the location context from GPS logging, the body acceleration can provide unprecedented details about the physiological and health state of an individual. Wilson et al. (2014) showed that even the past medical history of people can be detected via the fine-scale reporting of 3D-acceleration vibrations (Wilson et al. 2014; Sands et al. 2015). Similarly, the sickness behaviour of animals—perhaps one of the best remote measurements of health in wildlife—can be inferred from 3D-acceleration loggers (Wilson et al. 2014). Even more so, stressful situations generally change the way bodies are moving, which again is easily detected using 3D-acceleration sensors. Ideally, other sensors that help to fine-position a body in space such as gyroscopic, magnetic, and pressure sensors can be combined with 3D-acceleration sensing to achieve a near-perfect reconstruction of the spatial state of an individual at critical times during its life (Wilson et al. 2014; Williams et al. 2017). Such IMU (inertial measurement unit) technology is already highly developed in commercial fields such as drone flight and is now adapted to physiological wildlife studies (Floreano and Wood 2015). 3D-acceleration logging also allows a first glimpse into the energetics of an individual via the calculation of ODBA, the overall dynamic body acceleration (Wilson et al. 2006; Halsey et al. 2011). Although it is only a crude first estimate of the detailed energy expenditure of an individual, ODBA is very helpful in understanding long-term differences in energy use between species, populations, and individuals, as well as in individuals over time and space. As a very helpful tool in conservation physiology, it is now possible to construct ‘energetic landscapes’ to understand which locations within the movement range of an individual demand more power from the animal than others (Scharf et al. 2016).

The internal state of an individual is perhaps best approximated from body temperature and heart rate logging (Butler et al. 2002, 2004). Based on Fick’s equation, heart rate can provide a good proxy for true energy expenditure of an individual if the stroke volume is constant or known, and if the oxygenation of the blood does not change much (Halsey et al. 2008). Combined with ODBA measurements, the quantification of heart rate provides a rather comprehensive assessment of instantaneous energy expenditure, stress, and movement energetics (Halsey et al. 2009; Clark et al. 2010; Duriez et al. 2014). The concurrent quantification of body temperature adds an important component of thermoregulation (potential heat and cold stress), but—depending on context—may also indicate a disease or an immune response (Adelman et al. 2010, 2014).

In the future, bio-logging tags that possess all above-mentioned sensors will become common and be miniaturised massively (Wikelski et al. 2007; Bridge et al. 2011). Additional features in tags will include on-board cameras that are triggered by pre-set (previously observed and internally programmed) behavioural changes to also allow researchers to study the social context of individuals, both within conspecific groups as well as during interspecific interactions (Watanabe and Takahashi 2013; Kane and Zamani 2014; Hays 2015). Understanding the collective behaviour of individuals in the wild is of great importance as we appreciate more and more that physiological changes during the life history of individuals are strongly influenced by biological interactions (Chikersal et al. 2017). Another aspect of the movement physiology of animals that can soon be quantified better is the relative positioning of limbs or other body parts relative to each other. Body area networks of sensors that communicate with each other through bluetooth-like radio connections may help better understand the forces exerted and received by individuals (Ullah et al. 2012). What is still in its infancy are data loggers reporting olfactory information produced or received by free-roaming animals (Gagliardo et al. 2013; Reynolds et al. 2015; Wikelski et al. 2015). Nevertheless, some progress is being made in the atmospheric chemistry field to miniaturise such sensors into autonomous data loggers that could eventually be used to study olfactory components of the life of individuals. In the future, sound and video could also be recorded by loggers to better apprehend the environmental context around the focal animal to better explain its behaviour.

Although we can use the sensing system mentioned above to infer the internal physiological state of individuals in many cases, there is still a major lack of technological developments applying the existing physiological sensors to work autonomously in free-roaming animals (Ropert-Coudert and Wilson 2005; Ponganis 2007; Cooke et al. 2014). In human and veterinary medicine many sensors already exist that could potentially be combined with bio-logging devices; however, there is still little concrete progress in this field with some notable exceptions (Schobel et al. 2013). Such sensors could detect glucocorticoid or other hormonal breakdown products, blood sugar, or blood oxygenation, and stroke volume (Chen and Chatterjee 2013; Turner 2013). Nevertheless, physiological ecologists will always carefully consider if a correlate of a physiological state is sufficient to provide the answer to an eco-physiological question, particularly in the context of conservation physiology (Cooke et al. 2014; Wilson et al. 2015; Lennox et al. 2016).

Great progress has also been made in terms of neuro-logging devices (Kang et al. 2016) that report the state of an animals’ brain or neuronal systems while the animal goes about its daily business. Starting from homing pigeons, the brain activity during complex navigational tasks has been addressed (Latanov et al. 2005; Vyssotski et al. 2009). The study of sleep in wild animals is also progressing rapidly (Rattenborg et al. 2008b; Voirin et al. 2014) with, sometimes, spectacular insights into the physiological functioning of individuals in their natural context (Rattenborg et al. 2016).

Compared to the internal physiological or neurological state, it is now rather straightforward to annotate the environmental context along the path of an individual in space and time (Shamoun-Baranes et al. 2010). As the satellite and in situ remote sensing systems have become public and much more generally available recently (Turner et al. 2015), there even exist automated environmental annotation facilities such as the Env-Data Module in Movebank (Kranstauber et al. 2011; Dodge et al. 2013). Using these annotation systems it is now easy and straightforward to determine dozens of environmental factors potentially influencing the behaviour and physiology of wild animals on the move. Here, we provide an example of such an analysis for the annual movement of Himalayan vultures. Moreover, as the remote sensing system can also provide the information of environmental factors in the close and far vicinity of the individual, we can now determine which environments the individual could have moved through, but did not visit. Knowing what environmental factors an individual avoids can be even more informative than knowing those that it uses (Safi et al. 2013; Kranstauber et al. 2015). In addition, we also start to learn—by the apparent mistakes in environmental selection an individual makes—what individuals know about their environment, and what they may not know (Cagnacci et al. 2010).

## Case study: Himalayan vultures

Himalayan vultures are the heaviest flying vertebrate scavengers in Asia. Here, we showcase the use of such bio-logging devices to study the conservation physiology of Himalayan vultures *Gyps himalayensis*, which share their conservation fate with many large and long-lived vertebrate species around the world that are in serious decline or on the brink of extinction [for vultures, see: (Ogada et al. 2012; Buechley and Şekercioğlu 2016)]. Vultures are particularly threatened: of the world’s 23 vulture species, 12 are critically endangered or almost extinct (Mandel et al. 2008; Dodge et al. 2014; Ogada et al. 2015). In the Asian biomes, 4 species of vultures are critically endangered and 4 are in lower threat categories (IUCN 2017).

Survival threats to global vulture populations are from poaching and indiscriminate poisoning, notably by the use of non-steroidal anti-inflammatory drugs (NSAIDs) in veterinary practises of livestock farming (Proffitt and Bagla 2004). In the Hindu state, livestock corpses (cows and oxen) are left for wildlife-predatory birds and mammals, and scavengers for consumption. At the general surprise, after having been considered as the most numerous raptors in the world, vulture populations in India were decimated, with 98% population decline relative to 1980s (Virani et al. 2011). The mortality of vultures in India was caused by renal failure and visceral gout, as a result of feeding on livestock corpses administered with diclofenac (Swan et al. 2006). Despite prohibition of the use of some NSAIDs in livestock, vulture populations slowly recover but remain at extremely low levels (Prakash et al. 2012; Galligan et al. 2014), with an exception of the three highland species (Bearded vulture, Griffon vulture, and Himalayan vulture) which apparently still enjoy relatively stable populations, but may face similarly serious declines within the next three generations. Diclofenac is banned from use in livestock in India, Nepal, Bangladesh, and Pakistan, and the use of ketoprofen in Bangladesh is prohibited. Gyps species are captive bred and raised to be released in “Vulture Save Areas” starting at the end of 2017 or during 2018, as an effort towards vulture population recovery and stabilisation. Emergence of new threats from the use of nimesulide—a NSAID substitute for diclofenac—is also known to cause mortality in all *Gyps* species (Cuthbert et al. 2007; Acharya et al. 2009). Recently, the Himalayan vulture is listed as “near-threatened” by IUCN, but a recent study proposed to update it as “vulnerable” with the prospect of population decline in the next three generations (Paudel et al. 2015). Beside their diet, two aspects of their lifestyle make vultures particularly vulnerable to human threats: their slow life cycle [a low reproductive rate, delayed maturity, and long lifespan make vulture demography highly sensitive to any increase in adult mortality (Ricklefs and Wikelski 2002)], and their movement ecology. Most vulture species perform long distance movements during their daily foraging routine, and many species are migratory or erratic at some stage of their life cycle (Dodge et al. 2014). These large scale movements complicate the conservation actions usually applied at small scale (e.g., in a reserve or a national park) and justify the need to study their movements using bio-logging devices to understand the reasons of their movements and plan adequate conservation actions at the appropriate scale (Wilcove and Wikelski 2008).

In our study, we try to find the specific, individual causes of mortality of some Himalayan vultures using bio-loggers with special emphasis on their annual movement strategies and the environmental conditions encountered by the birds during the course of their movements. We concentrate on immature and juvenile birds because young and naïve birds are also generally engaged in erratic movements, potentially leading them to dangerous areas (López-López et al. 2013; Phipps et al. 2013).

## Methods

### Bird capture and data recording

18 non-breeding Himalayan vultures were captured in Bhutan between November 2014 and February 2015 using wire mesh–mesh cage traps, weighed and measured. All birds included in the study were immature individuals at an estimated age of 1–2 years. Individuals were equipped with high-resolution GPS data loggers (45 g, cell phone link, e-Obs GmBH), using an approximately 30 g Teflon-nylon harness. Birds were then filmed before release to calibrate their behaviour against 3D-acceleration data that were collected by the tags (see below). Daily between 02.00 and 20.00 hour UTC, loggers were set to periodically collect 1 Hz GPS fixes for 10 min, whenever solar charge allowed. Whenever there was low sunlight charge of the tag, the GPS fix rate was lowered to 30 min. We collected a total of 2,923,736 GPS fixes between February 1, 2015 and January 31, 2016. The tags were also set to collect 3D-acceleration data at 15 Hz per axis for a duration of 4 s at 2 min intervals throughout the day. These data were also transmitted through the GPRS connection of the tags. Data are available through the Movebank archive (https://www.movebank.org/node/15294).

### Seasonal home range, initial movement distance and migration direction

We determined the home ranges of individuals for the winter period (November–April) and summer period (June–September). We used three kernel density estimates (50, 95, and 99%) and showed the 95% estimated home range in our maps. Home range area statistics were computed only for the stationary phases of the annual cycle, using Hawthtools with ArcGIS10.0 fixed-kernel estimators at a fixed interval >10 min, to avoid strong autocorrelation of positions. When the birds moved away from their winter ranges, we determined the initial movement distance as great circle distance from the centroid of the winter range towards the centroid of their first staging area, i.e., an area where they spent more than a week. We then determined the migration direction as the compass direction between these two points.

### Environmental data annotation, acceleration analysis, and circumstances of disappearance

In our analysis we focused on non-breeding individuals (*N* = 18) as they all face the same life history constraints, i.e., the survival analysis is not confounded by the potentially differing demands of reproduction. GPS tracks and ACC data transmitted from bio-loggers on Himalayan vultures in the field are stored in the Movebank online database (Fiedler and Davidson 2012). We annotated 13 environmental factors (SRTM elevation, ECMWF Interim Full Daily Pressure Level Pressure Vertical Velocity, ECMWF Interim Full Daily Pressure Level Relative Humidity, ECMWF Interim Full Daily Sunshine Duration, ECMWF Interim Full Daily Total Precipitation, GlobCover 2009 2009 Land Cover Classification, Movebank Orographic Uplift from ASTER DEM and ECMWF, ECMWF Interim Full Daily Pressure Level V Velocity, ECMWF Interim Full Daily Pressure Level U Velocity, ECMWF Interim Full Daily Pressure Level Temperature, SEDAC GRUMP v1 2000 Population Density Adjusted, MODIS Land Terra Vegetation Indices 05 deg Monthly NDVI, Movebank Thermal Uplift from ECMWF) to our GPS data using an automated Env-DATA System available in Movebank (Dodge et al. 2013).

These environmental data were annotated in space and time and interpolated as needed to the GPS data.

We used the 3D-acceleration data to determine the behaviour of the birds throughout their annual cycle. For this, we plotted the data using the Movebank acceleration-viewer annotation extension (https://www.movebank.org/node/5920) and visually annotated them from our observation and photograph-calibrated 3D-acceleration data, prior to the release of the birds in a wire mesh cage trap where they were feeding, preening, sitting, and interacting with other vultures (for similar methods, see also (Resheff et al. 2014). Combined with the GPS data that gave the spatial and movement (speed, height, and directional heading) context, we used the behavioural classes resting, flying (further distinguishing between flapping flight and gliding flight), and forage to characterise the birds’ actions throughout the year. For the 5 birds that died during the observation year, we detailed the location and acceleration information within the last two weeks of their disappearances. We also sent field teams to three of the carcasses to confirm the most likely cause of death.

### Statistical analysis

We used statistical package SPSS 22 for analysis. We included one entire year of data, starting from February 1, 2015, for all birds except for the five birds that died. For the dead birds we did not include the last two weeks before death into our analyses to ensure that we do not bias the mortality analysis by factors that preceded death (e.g., if birds that sit on the ground continuously are more likely to be poisoned or killed by hunters). For each bird we calculated, for its entire lifetime (minus the last two weeks), or for a maximum of one year, average values for their behavioural parameters (such as flight altitude above the surface, % time resting, % time thermalling, etc.) and the associated environmental parameters (such as NDVI, temperature, humidity, etc.), at each GPS position at a minimal time interval of 10 min. Whenever we calculated percentage data, we arc-sin transformed them for the statistical analysis. For the analyses of environmental or behavioural data, we included all parameters that were not correlated stronger than ±0.2. To come up with hypotheses as to which behavioural or environmental factors were potentially influencing the survival of individuals, we conducted several independent sample *t* tests. We are aware that the overall low sample size of our data set does not allow for robust statistical analyses with regard to the multitude of factors, as well as their interactions, that may affect survival. However, as a first approach to determine factors that might affect survival in Himalayan vultures, these analyses may be valid.

## Results

### Seasonal home range, initial movement distance, and migration direction

**Fig. 1:**
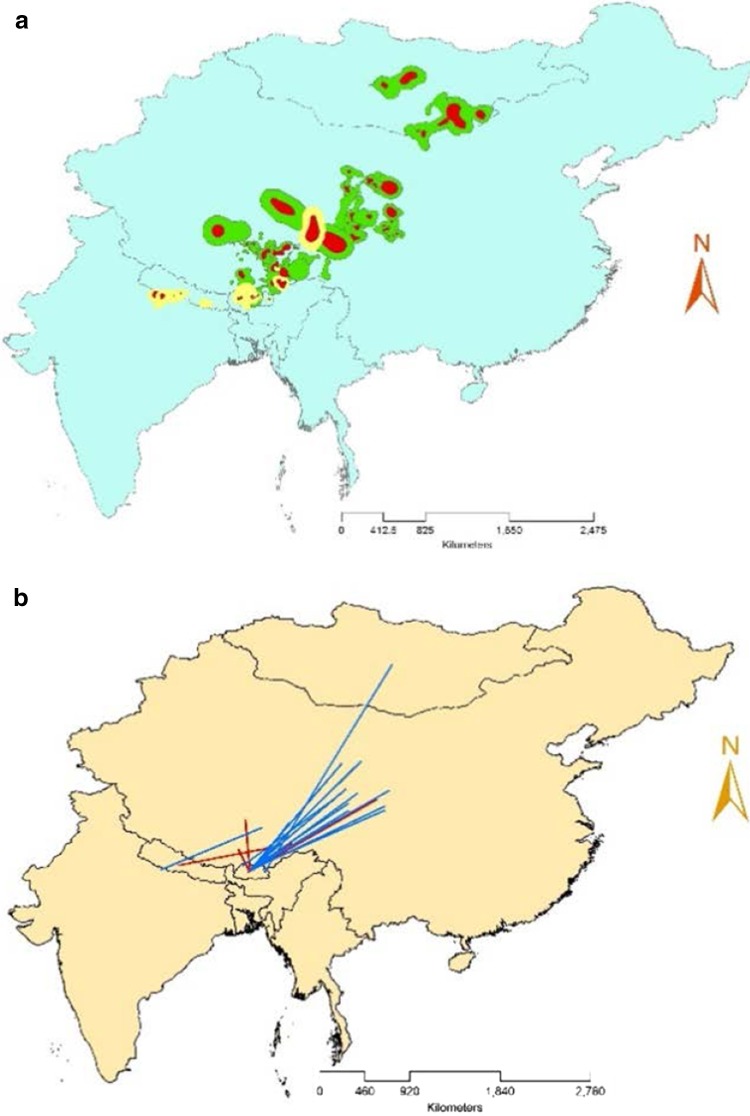
Annual movement characteristics of Himalayan vulture (*N* = 18) (**a**). Seasonal home ranges: *Pale yellow* polygons (95% convex) with *red* polygons (50% convex) in the centre represent the extent of the winter home range, covering India, Nepal, and Tibetan Autonomous Region (China). *Green* polygons (95% polygons) with *red* polygons (50% polygons) in the centre denote the extent of the summer home range, covering Mongolia, Inner Mongolia, and China, in Asian landscape (**b**). Initial movement distance: Each *line* symbolises a departure direction and distance from the wintering areas (India, Nepal, and Bhutan), northward to the first staging area during migration to the summering areas. The *red lines* are of Himalayan vultures that died during their first observation year, *blue lines* indicate birds that survived their first year of observation

The observed birds migrated extensively both latitudinally as well as altitudinally, moving from the highest altitudes of the Himalayas to low altitude range lands in India. In winter, they descended to lower areas in the northern plains of India, the Himalayas of Nepal and Bhutan, and the Himalayan Plateau and south eastern part of China (Fig. 1a). The mean winter home range covered 13,973 km^2^ ± 6507 SE (*N* = 18, 95% CI = 1220–26,727 km^2^). In summer, Himalayan vultures ascended to the highlands of the Himalayan Plateau, Inner Mongolia, and Mongolia. The mean summer home range (61,130 km^2^ ± 20,062 SE, 95% CI = 21,809–100,452 km^2^) was approximately four times larger than the winter home range (Fig. 1a).

Migrant Himalayan vultures crossed the Himalayan mountain range biannually during their north and south bound migrations. During the migration, they made stopovers at favourable staging areas. Here, we measured the initial movement distance between the centres of their first major staging areas and their wintering areas for the first northward migration of the year (Fig. 1b). The mean initial movement distance was 1086 km (±119 SE, 95% CI = 892–1319 km), covered in a maximum of two weeks. The departure direction of Himalayan vultures was predominantly north-easterly (Fig. 1b), with a median departure direction of 52.5° (95% CI = 34.58°–98.88°) during their winter-to-summer migration.

### Ultimate causes of mortality

**Fig. 2:**
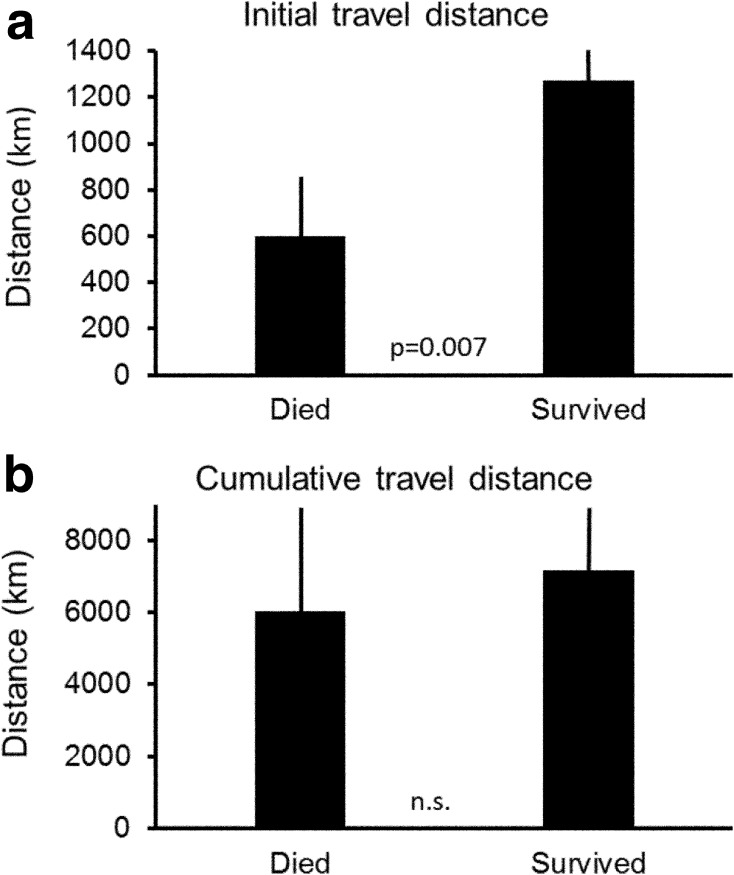
**a** The initial travel distance (from the wintering and the first stopover site) was shorter in individuals that died during their first observation year. **b** The cumulative travel distance did not differ between individuals that died or that survived. Data show means ±95% confidence interval, *p* values from *T* test

During one year, 13 of 18 birds survived while 5 died during their migration movements from the winter areas to the summer areas. Birds that died used as first stopover a site located at a twice lower distance than birds that survived (Independent samples *t* test, *F* = 0.08, *t* = −3.1, *df* = 16, *p* = 0.007; Fig. 2a). However, the birds’ survival did not depend on the total distances they moved (Independent samples *t* test, *F* = 0.55, *t* = −0.8, *df* = 16, *p* = 0.43; Fig. 2b). These data suggest that surviving birds had a more straight path towards the summer areas in comparison to birds that died, who had a more sinusoidal path and stopped several times before reaching the summer grounds. Thus, we conclude that the initial straight-line distance from the wintering sites to the first staging areas mattered for the survival.

To assess the environmental and behavioural parameters that could affect individual mortality, we selected 7 individual performance variables of the vultures: flight altitude above the surface, absolute flight height, proportion of sitting on the ground versus flying, proportion of flight in a straight line versus thermalling (circling) flight, and the strength of the thermal uplift or of orographic uplift (as calculated from the Movebank data models). We also included 7 environmental variables: sunshine duration, precipitation, NDVI, ambient temperature, relative humidity, northerly winds versus easterly or westerly winds, as well as one anthropogenic parameter (population density) in the analysis.

**Fig. 3:**
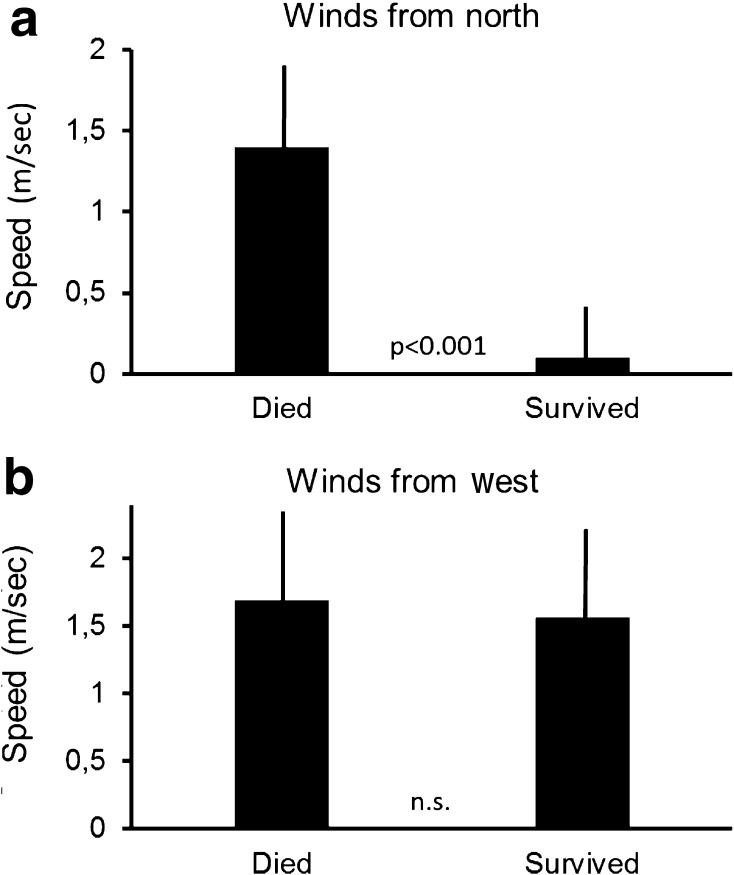
**a** Animals that died had on average stronger headwinds from the north (meridional winds) during their first migratory bout. **b** The winds from westerly or easterly directions (zonal winds) had no effect on survival fate. Data show means ±95% confidence interval, *p* values from *t* test

**Fig. 4:**
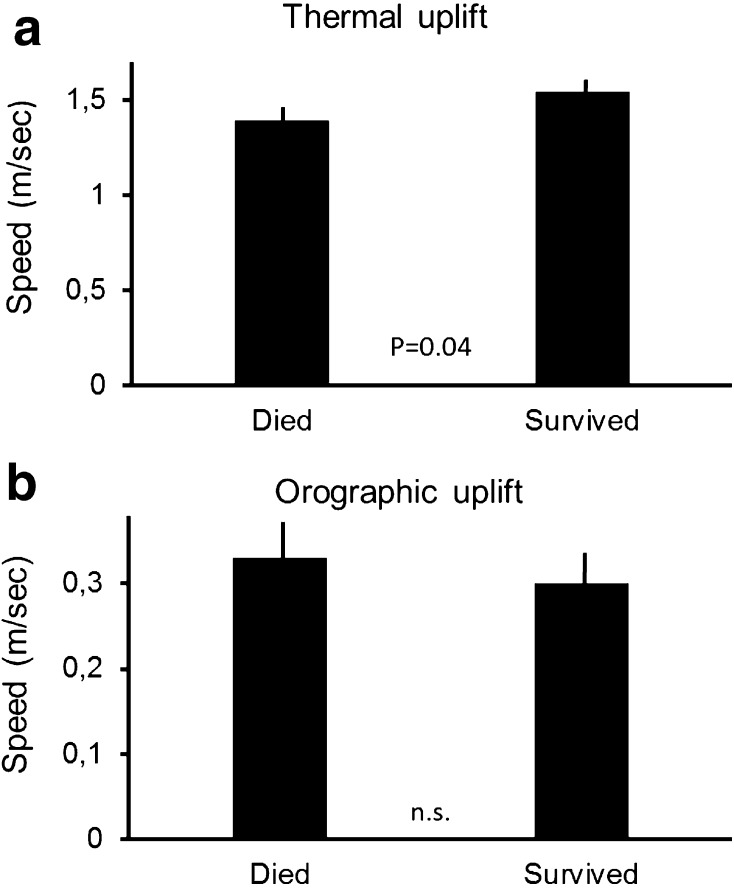
**a** Vultures that survived selected areas with stronger thermal uplifts compared to individuals that died. **b** The flights in orographic uplifts did not differ between individuals that died or those that survived. Data show means ±95% confidence interval, *p* values from *t* test

**Fig. 5:**
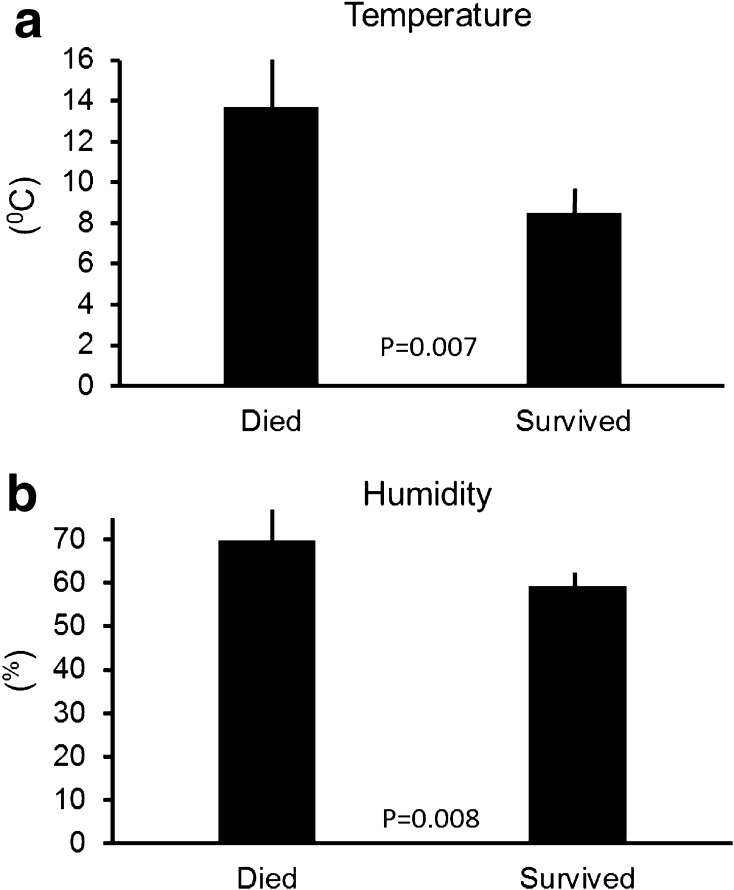
**a** Vultures that died selected areas with higher ambient temperatures compared to individuals that died, and also selected areas with higher relative humidity (**b**). Data show means ±95% confidence interval, *p* values from *T* test

**Fig. 6:**
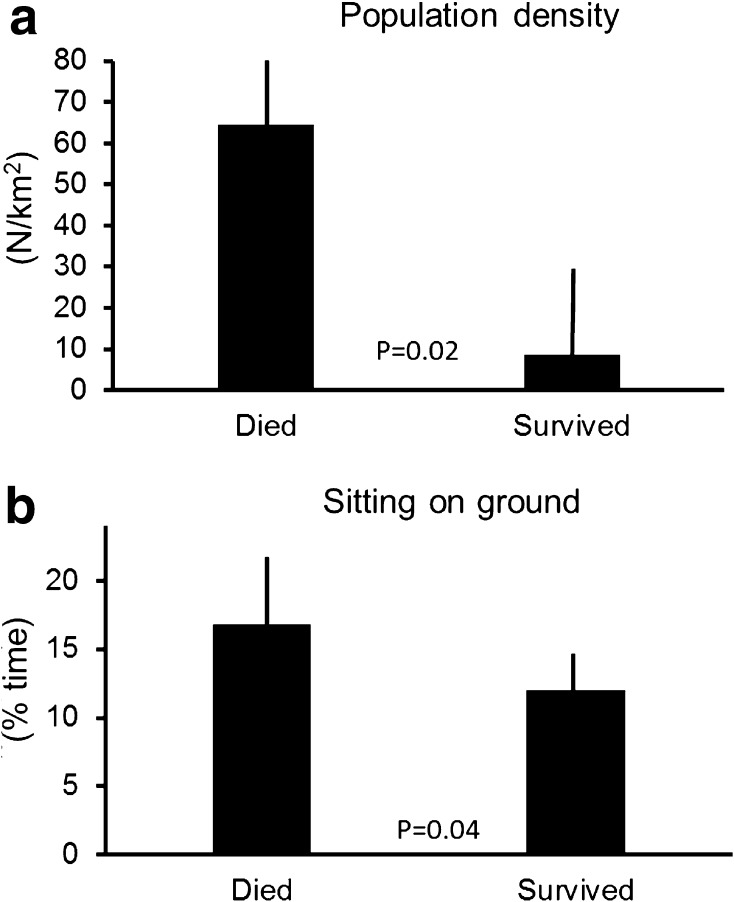
**a** Vultures that survived selected areas with much lower population density compared to individuals that died. **b** Surviving vultures sat much less on the ground compared to vultures that died. Data show means ±95% confidence interval, *p* values from *t* test

Vultures that often flew against headwinds from the north did not survive their first year (Independent samples *t* test, *F* = 0.27, *t* = 3.4, *df* = 16, *p* < 0.001; Fig. 3a). In contrast, how individuals used winds coming from easterly or westerly directions did not matter (Independent samples *t* test, *F* = 0.39, *t* = 0.26, *df* = 16, *p* = 0.79; Fig. 3b). Vultures which were less able to find thermal updrafts were more likely to die, while individuals which were able to find and harvest thermal updrafts survived (Independent samples *t* test, *F* = 0.66, *t* = −2.0, *df* = 16, *p* = 0.04; Fig. 4a). In contrast, the influence of orographic uplift on survival was not significant (Independent samples *t* test, *F* = 0.23, *t* = 0.95, *df* = 16, *p* = 0.34; Fig. 4b). Himalayan vultures that stayed most of the year in relatively cold places were more likely to survive (Independent samples *t* test, *F* = 2.9, *t* = 2.77, *df* = 16, *p* = 0.007; Fig. 5a). At the same time, individuals that stayed in drier areas were more likely to survive (Independent samples *t* test, *F* = 0.07, *t* = 2.7, *df* = 16, *p* = 0.008; Fig. 5b). Birds that, throughout the year, spent most of their time in areas with high human population density were more likely to die (Independent samples *t* test, *F* = 17.9, *t* = 2.38, *df* = 16, *p* = 0.02; Fig. 6a). However, NDVI was not a decisive factor in the survival of Himalayan vultures (Independent samples *t* test, *F* = 0.4, *t* = −1.14, *df* = 16, *p* = 0.26), indicating that individuals survived better if they chose areas with high plant productivity (high NDVI) that at the same time had low population densities. Such areas can be found in the inaccessible highlands of inner Mongolia and parts of Tibet, but also Mongolia. However, the percentage of the time focal individuals sat on the ground (resting) significantly influenced their survival: vultures that spend more time on the ground (excluding the two weeks before individuals died), instead of flying, were more likely to die (transformed data: Independent samples *t* test, *F* = 9.7, *t* = 2.01, *df* = 16, *p* = 0.04; Fig. 6b). Thus, it is apparently beneficial for the survival of vultures to spend as much time in the air as possible.

Environmental factors that we also considered to potentially affect survival, but that apparently did not influence the survival of Himalayan vultures were: flight altitude above the surface (*p* = 0.09), absolute flight height (*p* = 0.41), sunshine duration (*p* = 0.08), precipitation (*p* = 0.17), or the proportion of time flying via thermalling (*p* = 0.18) or via straight-line flight (*p* = 0.31).

### Proximate causes of mortality

The five birds that died in the first year of observation were Palya, Dorje, MaaJoen, JigDor and Baen (see data in DOI to be provided upon publication). Using the high-definition data loggers, we could identify the most likely immediate causes of death in these 5 individuals. Palya presumably died from poisoning because along with it there were 7 other dead bodies of vultures. The tag was found on the ground and retrieved (Yachang Cheng, pers. obs.). Dorje, MaaJoen, and Baen disappeared in areas with human population and their demise was presumably linked to human causes. Baen’s acceleration record showed it may have died from sickness: the acceleration showed highly irregular activity towards the days of her death. JigDor’s death in Nepal was possibly linked to the ingestion of diclofenac. Nepal is a Hindu state where dead cows are not consumed. Apparently JigDor had fed by the riverbed next to an agricultural field and had flown far away from the feeding site to a place where it soon died.

## Discussion

High-definition bio-logging of the annual movements of a cohort of immature Himalayan griffons highlighted the environmental and individual factors responsible for their survival. Although only a subset of parameters that, in principle, can be recorded electronically was in fact monitored in these individuals, the high-definition location data in relation to remote sensing data from earth observation satellites allowed us to pinpoint areas of success or failure in these individuals. Such data can be highly relevant for conservation as they show exactly what the cause of death is in a group of animals that is globally endangered. Similar studies have successfully been done in other raptor species during their migration (Klaassen et al. 2014). We also want to caution that our analysis is still using long-term averages of environmental parameters to allow for an overall comparison between individuals. In future analyses using a larger sample size of individuals and ideally a lifetime tracking approach, it will be important to determine seasonal and regional differences in how both behavioural and environmental parameters influence survival.

The factors that turned out to be very important were long range movements away from their wintering quarters, especially in areas in India and Nepal that have high human population densities (Acharya et al. 2009). Himalayan vultures survived particularly well in areas that were not dominated by humans but still have enough vegetation (or high NDVI) and thus provide carcasses of vertebrates that supply food for the vultures. Apparently, vultures mostly feed on livestock and not wildlife carrion. Thus, desert areas are not providing good food supplies, whereas areas with intermediate NDVI appear to be better. We suggest that villages with rural communities could be the most appropriate location to find dead livestock carcasses (if they can find them before farmers, or at sites with sacred cows, not eaten by people), and sky burial sites.

We did not yet include the birds’ time spent in protected areas in the analysis, but future studies will address this aspect.

Our data also highlighted individual capacity as a decisive factor for survival. Immature vultures that were finding thermals at a better rate and were better able to exploit tailwinds had a higher survival probability. We would like to emphasise that the calculation of thermal and orographic updrafts is obviously complicated within mountainous regions such as the Himalayas, thus, our analysis can only provide a suggestion into a possible relationship between updraft components and survival. In the future, such relationships will be entirely testable with more refined environmental data. In Eurasian griffon vultures, it was shown that young birds behaved less efficiently in thermals under strong winds compared to experienced adults (Harel et al. 2016). This is similar to what has recently been found in young storks (Flack et al. 2016; Rotics et al. 2016) and shows how important the ontogeny and ontogenetic experience is for the survival of juvenile birds. It should also be noted that many of the foraging areas of the Himalayan vultures are linked to human habitation directly, namely the sky burial sites. We estimated that approximately 20% of their foraging activities in China (Tibet, Inner Mongolia) were at traditional sky burial sites. This is consistent with estimates just based on population numbers by Ming (pers. comm.).

The specific causes of death included starvation and poisoning, which was confirmed on the ground in two of the five cases. The use of acceleration data combined with GPS tracks collected in the two weeks before death was useful to give cues about the likely cause of death. Thus, even for the Himalayan vultures that are much less exposed to diclofenac poisoning, the apparent poisoning of mammalian carcases may turn out detrimental for the survival of these birds (Acharya et al. 2009; Das et al. 2011).

With the rapid technological advancement and miniaturisation of devices in animal tracking as well as the inclusion of multiple sensors (such as high-resolution GPS and ACC, pitot pressure, gyroscope, magnetometer) we can now track annual movements and soon the entire life history of an animal in high-definition. In the future, animals outfitted with new generation tags may become sentinels of weather and climate as well as prevailing environmental conditions at the finest spatial and temporal granularity (Kays et al. 2015). Tagged animals will collect and relay real time information of environmental conditions, surrounding tracked animals or record ephemeral environmental conditions quite precisely. Once such systems are implemented, our interpretations of animal behaviour as well as the challenges faced by animals in the wild in relation to environmental factors (weather, habitat, and land use) may become even easier. As a result, we may be able to intervene in the species conservation management in real time at the very location where animals face survival challenges (Wikelski and Cooke 2006; Cooke et al. 2014). Such bio-logging devices combining GPS and ACC data transmitted by GSM could help finding and rescuing several vulture species (*G. barbatus, A. monachus, N. percnopterus*) that are the focus of reintroduction or rehabilitation programmes in France (O. Duriez, unpublished results).

Remote sensing environmental data (weather, habitat, and human land use) can now be commonly used in animal tracking. For example, the global data repository Movebank (Fiedler and Davidson 2012) offers open access environmental data that can be easily annotated to GPS data with the Env-DATA system (Dodge et al. 2013). In our analysis, 13 environmental variables were used in the survival model. While several environmental parameters significantly contribute to the survival and death of Himalayan vultures, NDVI parameters failed to provide evidence of significance. While it is intuitive that areas with high NDVI mean greater biodiversity, it may not necessarily mean high ungulate populations, which Himalayan griffons are dependent upon for survival. Survival of vultures around the globe is vehemently threatened by poisoning, poaching, and use of NSAIDs in husbandry practises of livestock farming (Ogada et al. 2012). Advanced bio-logging may help to conserve them and at the same time provide a new way to study the physiology of life histories in the wild (Wikelski and Ricklefs 2001; Block 2005).

## Abbreviations

3D: Three dimensional
ACC: Acceleration
ECMWF: European centre for medium-range weather forecasts
GPRS: General packet radio service
GPS: Global positioning system
GSM: Global system for mobile communications
Hz: Hertz
IMU: Inertial measurement unit
IUCN: International union for conservation of nature
MODIS: Moderate-resolution imaging Spectroradiometer
NDVI: Normalized difference vegetation index
NSAID: Non-steroidal inflammatory drugs
ODBA: Overall dynamic body acceleration
SRTM: Shuttle radar topography mission
UTC: Coordinated universal time
